# Meta-analysis of substitution value of maize with cassava (*Manihot esculenta Cratnz*) on growth performance of broiler chickens

**DOI:** 10.3389/fvets.2022.997128

**Published:** 2022-11-14

**Authors:** Ifeanyichukwu Princewill Ogbuewu, Christian Anayo Mbajiorgu

**Affiliations:** ^1^Department of Animal Science and Technology, Federal University of Technology, Owerri, Nigeria; ^2^Department of Agriculture and Animal Health, University of South Africa, Pretoria, South Africa

**Keywords:** *Manihot esculenta*, poultry, growth traits, data synthesis, meta-regression

## Abstract

There are variable results on the effect of cassava on the performance characteristics of broiler chickens. As a result, this meta-analysis was performed to determine the effect of cassava on feed intake, feed conversion ratio (FCR), and average daily gain (ADG) in broiler chickens. A methodical search performed on Google Scholar, Scopus, Web of Science, and PubMed databases as well as individual journals yielded 365 published articles. Out of 365 studies, 23 that met the inclusion criteria were used for the meta-analysis. Outcome measures were pooled using a random-effects model. Results were expressed as standardized mean differences (SMD) at 95% confidence intervals (CIs). Subgroup and meta-regression analyses were used to explore the effects of studied covariates (broiler strain, inclusion level of cassava, number of broilers per replicate, cassava processing methods, and cassava form) on measured outcomes. Results indicated that cassava had a small positive effect on feed intake (SMD = −0.07, 95% CI −0.26, 0.12) and FCR (SMD = 0.14; 95% CI 0.82, 1.746), but a large negative effect on ADG (SMD = −1.67; 95% CI −1.99, −1.35) compared to the controls. Subgroup analysis by cassava form showed that wet fermented cassava peel meal (WFCPM) had a moderate impact on feed intake (SMD = 0.62, 95% CI 0.47, 0.77) and ADG (SMD = 0.66, 95% CI 0.37, 0.95) in broiler chickens compared with the controls. Our results also found improved growth performance in broiler chickens fed cassava at 4–10%. There is evidence of between-study variance, and studied covariates explain most of the sources of heterogeneity. This study concluded that the replacement of maize with 4–10% WFCPM improved growth performance traits in broiler chickens.

## Introduction

The high cost of maize in recent times has necessitated the search for close alternative energy feedstuffs for poultry production, especially in sub-Saharan Africa (1, 2). One of the largely used options was cassava (*Manihot esculenta*) (3–5). It is a woody shrub in the family *Euphorbiaceae*. Cassava is native to South America and is now found in abundance in tropical Africa, Southeast Asia, and Central America (6). Cassava is high in carbohydrates but low in proteins and essential nutrients (6), whereas the leaf is a moderate source of protein (6, 7). Cassava is low in protein and high in energy, although slightly lower than maize (4, 6). Cassava starch contains 17% amylose and 83% amylopectin, compared to maize starch, which contains 28% amylose and 72% amylopectin (8). The amylose content of maize is higher than that of cassava starch. Chauynarong et al. (6) found that cassava has more digestible starch than maize due to its higher amylopectin content. Cassava contains 2.55% crude protein, 27.75% crude fiber, 0.12% ether extract, and 1.70% ash on a dry matter basis (9). It also contains several minerals and vitamins. Compared to maize, the vitamin content of cassava is low. The mineral content of cassava root compared to maize is usually low, except for potassium (271 mg). Cassava leaves are low in energy and high in fiber and protein, whereas cassava peels are moderate in energy, low in protein, and higher in fiber than cassava tuber (10). It has been reported that fermentation can be used to improve the digestibility and nutritional quality of cassava and its by-products (5, 7).

Likewise, an important opportunity for animal nutritionists was to develop feed ingredients using by-products that would otherwise be unsuitable for human nutrition. This procedure may help to reduce the cost of animal feed while also contributing to environmental sustainability (11). Due to the absence of excellent post-harvest technologies, large amounts of cassava are wasted. Increased use of cassava in chicken diets will significantly reduce this wastage and also reduce the high cost of poultry feed. However, the utilization of cassava in the chicken diet is hindered by its low crude protein and amino acid content and the dusty nature of dried cassava meals (4, 5). Cassava has higher levels of cyanogenic glucosides (HCN), which limits its use in chicken feed (6). When such cassava is consumed by animals, an enzyme, β-glucosidase, which is produced by gut microbes, converts the HCN to hydrocyanic acid (12), which is toxic to animals. The ingestion of a high dose of HCN has been shown to inhibit the cytochrome oxidase of the respiratory chain (13). To minimize the adverse effect of hydrocyanic acid in the animal system, the liver and the erythrocytes produce two enzymes, thiosulphate cyanide sulfur transferase and mercaptopyruvate cyanide sulfur transferase, which are derived primarily from sulfur-containing amino acids like cysteine, cystine, and methionine. These enzymes combine with vitamin B12 to convert hydrocyanic acid into a harmless thiocyanate, which is then removed *via* the urine (13, 14).

According to Chauynarong et al. (6) and Omede et al. (7), these limitations on the use of cassava in chicken feed can be reduced by utilizing physical methods (sun-drying, pelleting, soaking, boiling, and mashing), biotechnological methods (wet and solid-state fermentation), and feed additive supplementation (amino acids, oil, and enzymes). Similar growth performance characteristics in broiler chickens fed whole cassava meals have been reported (15). Bhuiyan and Iji (16) found similar growth performance results in broiler chickens offered cassava with microbial enzyme supplementation. In contrast, Uchegbu et al. (4) found poor body weight gain in broiler chickens fed cassava fortified with palm oil. Other studies have found significantly reduced growth performance traits in broiler chickens fed cassava (17, 18). This wide variability may be due to differences in origin, parts of the cassava plant used, age of harvested crop, diet composition, and processing methods.

The use of systematic and explicit methods to identify, select, critically appraise, and analyse relevant published studies (19) in order to resolve conflict and increase statistical power has been advocated (20). Thus, this study aimed to evaluate the effect of the replacement of maize with cassava on the growth performance of broiler chickens through a meta-analysis using data from published articles. Sources of heterogeneity will also be assessed using subgroup and meta-regression analyses.

## Materials and methods

### Dataset development

**Figure 1:**
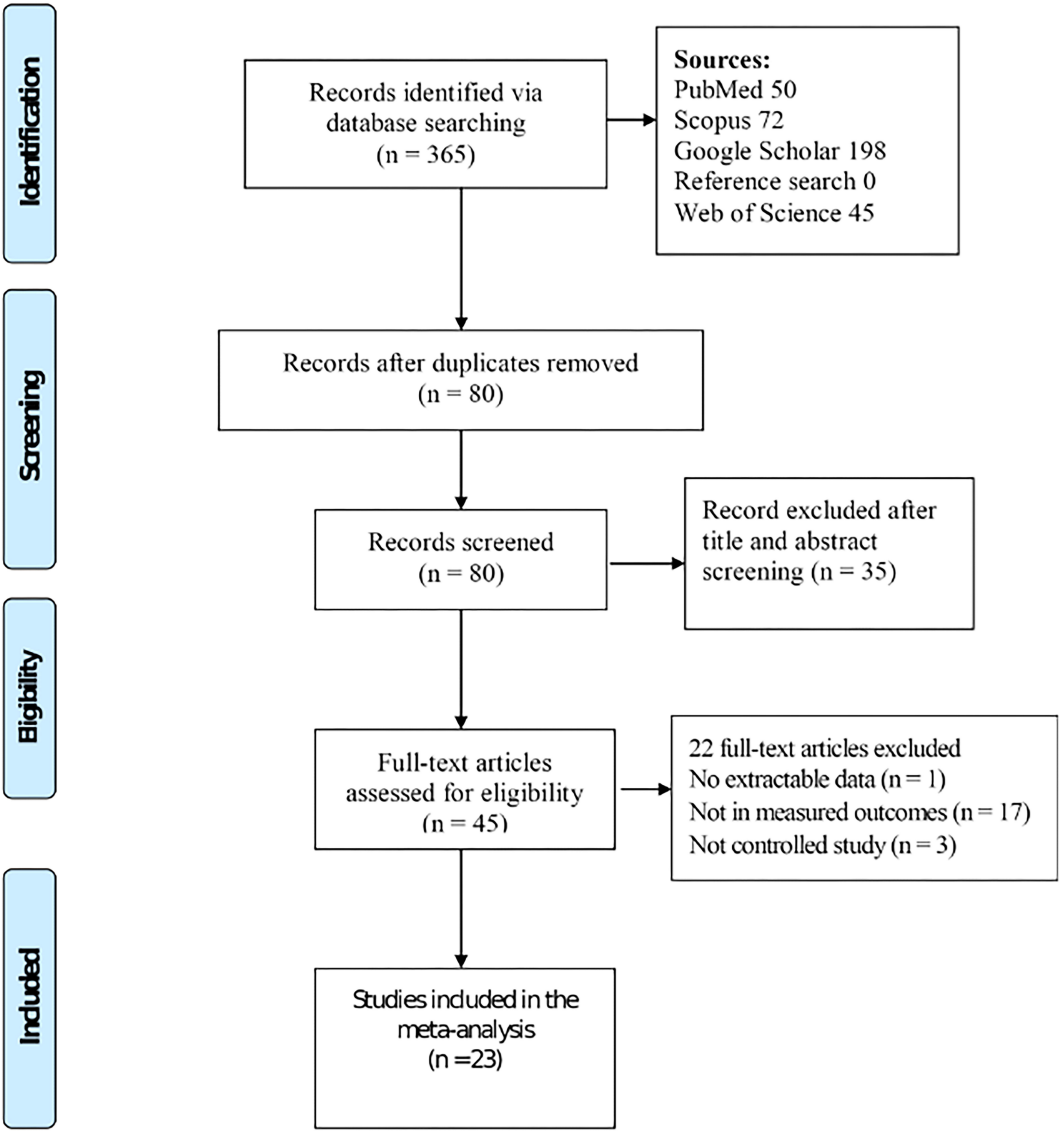
Study selection flowchart for the meta-analysis.

Articles were identified from a systematic search performed on PubMed, Web of Science, Scopus, and Google Scholar databases as well as individual journals using the keywords “cassava”, “broiler chickens”, “feed intake”, “FCR”, “feed conversion efficiency”, “body weight gain”, or “ADG” (21). Reference lists of retrieved articles were screened for relevant studies. Identified articles were independently reviewed for eligibility, and study inclusion debates were resolved *via* discussion and consensus. Selection criteria were based on the PICO framework, where *P* = **P**opulation (i.e., broiler chickens), I = **I**ntervention (i.e., dietary cassava), C = **C**omparison (i.e., diets with and without cassava), and O = **O**utcomes (i.e., feed intake, FCR, and ADG). Trials were included if (i) the study was peer-reviewed and published in English, (ii) the population was healthy broiler chickens, (iii) the intervention of interest was diets with and without cassava, and (iv) response variables of interest were feed intake, FCR, or ADG with their corresponding standard deviation (SD). Duplicates, articles with no extractable data, reviews, and non-broiler chicken studies were excluded. Non-controlled trials and studies that had not been published in English were also excluded. Following the Preferred Reporting Items for Systematic Reviews and Meta-analyses (PRISMA) guidelines, 23 articles were selected (Figure 1). The PRISMA checklist is presented in Supplementary Table S1.

### Data extraction

Authors extracted the following data from the 23 eligible studies: the surname of the first author, publication year, study country, study continent, rearing duration, covariates [cassava form, inclusion level, broiler strain, processing methods, and number per replicate (NPR)], sample size, SD, and response variables of interest (feed intake, FCR, and ADG) as shown in Supplementary Tables S2, S3. Data presented in the graphs were retrieved using WebPlotDigitizer (22). Studies that reported standard errors (SE) were converted to SD (23).

### Data analysis

All analyses were performed using Open Meta-analyst for Ecology and Evolution (OpenMEE) software (24). Effect sizes were presented as SMD at 95% CIs using a random-effects model. SMD was categorized as small (0.2), moderate (0.5), and large effect (≥0.8) using the classification method of Cohen (25). SMD was considered significant when the upper and lower 95% CIs did not include zero (20). The authors could not analyse the effect of rearing duration (starter vs. finisher) as a covariate in this study because of insufficient data. Subgroups with < 3 datasets were excluded from the study because of low statistical power. Publication bias was assessed using funnel plots and Rosenberg's failsafe number (Nfs). According to Jennions et al. (26), the results of a meta-analysis can be considered robust in the presence of publication bias when Nfs is > (5^*^n = number of comparisons + 10). Heterogeneity among studies was quantified using the chi-squared tests and I^2^-index developed from the Cochrane Q test according to Higgins and Thompson (27). The meta-regression analyses were performed on studied covariates to determine the amount of variability explained by the covariates. Subgroup analyses of covariates were performed to evaluate their effect on response variables. The meta-regression analysis was deemed statistically significant at *P* < 0.05. A mixed model was used to adjust the data in the meta-regression analysis using SMD as the dependent variable. The mixed-effect models were given as follows:


$$\begin{array}{l} \text{θ}i\;=\;\beta \;+\text{ β}i\;xij\;+\;\ldots \beta ip\;xip\;+\text{ μ}i \end{array}$$

where θi is the true effect treatment in the ith explanatory variable; β is the overall true effect cassava intervention; xij is the value of the jth variable (j = 1 2, …, p) for the ith explanatory variable; βi is change in the true size effect for a unit increase in the jth variable; and μi ~N (0 t^2^). The t^2^ indicates the heterogeneity that is not explained by the variable (28).

## Results

### Characteristics of included articles

A total of 365 studies were identified, with 23 of them meeting the pre-defined selection criteria (Figure 1). Studies used for the analysis were conducted in eight countries across four continents (Supplementary Table S2). Most of the experiments were performed in Nigeria. The earliest study that met our pre-defined inclusion criteria was published in 1988 (Supplementary Table S2). The most commonly used cassava form was cassava root meal (37.93%) followed by CPM (20.69%) as illustrated in Table 1. The most frequently reared strain was Cobb (28.57%), followed by Anak (23.81%) and Arbor Acres (19.04%).

**Table 1:** Definition of moderator variables included in the meta-analysis and their distribution matrix.

**Variables**	**Levels**	**Definition**	**Frequency (%)**
Cassava form	CRM	Cassava root meal	37.93
	CRM + ES	Cassava root meal + Enzyme supplementation	10.34
	CPM	Cassava peel meal	20.69
	CPM + CLM	Cassava peel meal + cassava leaf meal	6.89
	WFCPM	Wet fermented cassava peel meal	3.45
	SSFCRM	Solid state fermented cassava root meal	3.45
	CRSM	Cassava root sievate meal	3.45
	CRWM	Cassava root waste meal	3.45
	WFCRM	Wet fermented cassava root meal	3.45
	CPM + ES	Cassava peel meal + Enzyme supplementation	3.45
	SSFCPM	Solid state fermented cassava peel meal	3.45
Broiler strains			
	Cobb		28.57
	Ross		14.29
	Anak		23.81
	Arbor Acres		19.04
	Marshal		14.29
Inclusion level (%)			
	4–10		8.62
	11–20		31.04
	21–30		18.97
	31–40		20.69
	41–50		10.34
	51–60		10.34
NPR			
	< 10		36.36
	10		45.45
	>10		18.19
Processing methods			
	Sun-dry		74.19
	Sun-drying + ES	Drying + Enzyme supplementation	12.90
	WF + Sun-drying		6.45
	SSF + Sun-drying		6.45

### Feed intakes

**Figure 2:**
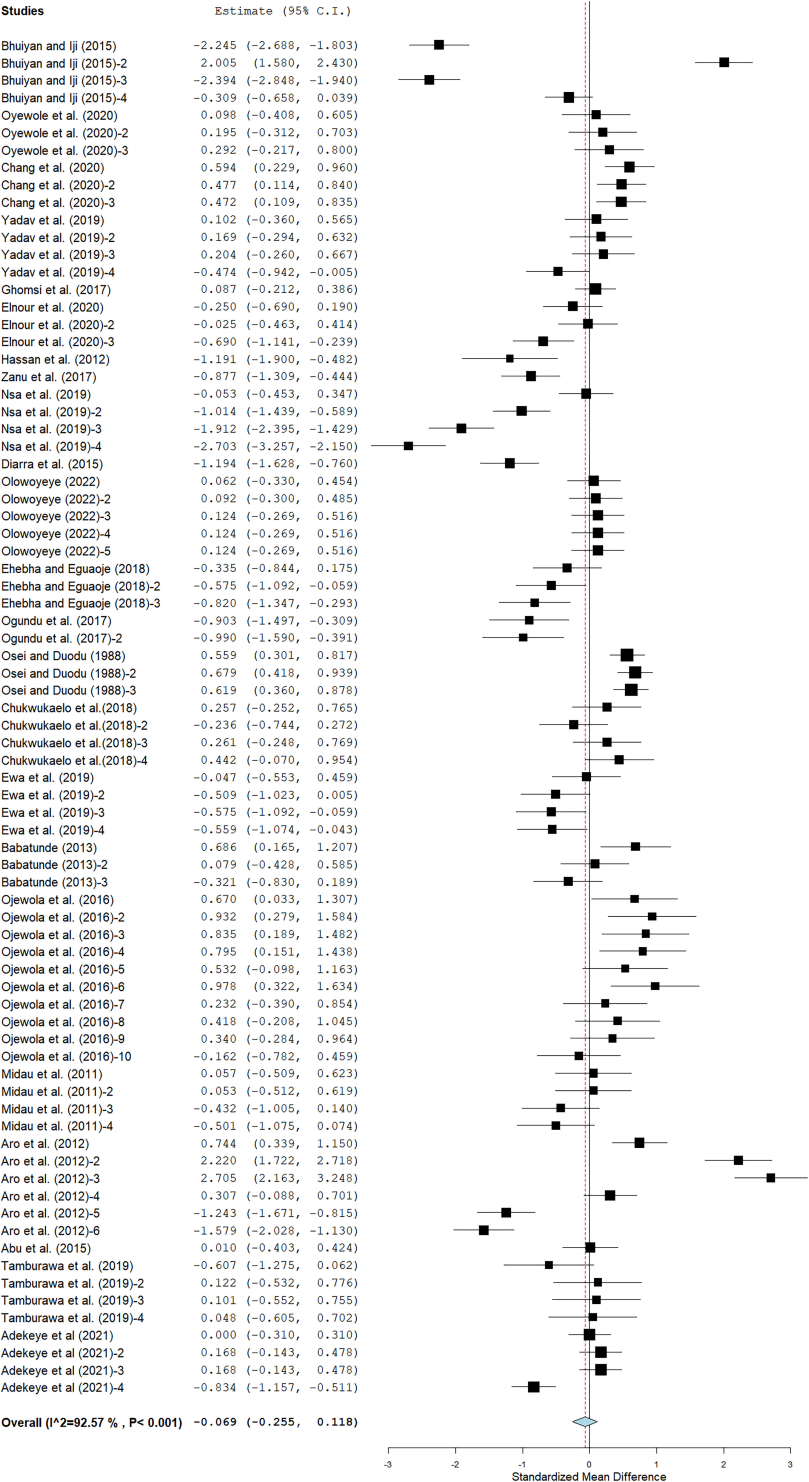
Modified error bar (MEB) of the impact of cassava on feed intake of broiler chickens. The thick vertical line is the line of no effect. Plots to the left of the solid vertical line indicate a decline in feed intake, whereas plots to the right mean an increase in feed intake.

The pooled results show that cassava had a small effect on feed intake (SMD = −0.07; 95% CI −0.26, 0.12; Figure 2). Subgroup analyses of the impact of covariates on feed intake are presented in Table 2. Subgroup analyses by cassava form suggested that WFCPM and wet fermented cassava root meal (WFCRM) had a medium effect on feed intake (SMD = 0.62; *p* < 0.001 and SMD = 0.55; *p* < 0.001, respectively). In converse, enzyme biodegraded CRM had a large negative effect on feed intake (SMD = −1.29; *p* = 0.034), whereas cassava root sievate meal (CRSM) had a moderate negative influence on feed intake (SMD = −0.42; *p* = 0.001). Cassava form had a moderate and non-significant impact on feed intake in Ross (SMD = 0.31; *p* = 0.331) and Arbor Acres (SMD = −0.59; *p* = 0.021). Mean effect sizes for inclusion level were found between 0.06 and 0.52, indicating that inclusion level had low to medium impacts on feed intake. Our subgroup results indicated that NPR had low to moderate effects on feed intake in broiler chickens. Results revealed that sun-drying as a processing technique had a moderate negative effect on feed intake (SMD = −0.24; *p* = 0.024). However, wet fermented plus sun-drying had a moderate positive impact on feed intake (SMD = 0.59; *p* < 0.001). There is evidence of substantial heterogeneity among studies included in the meta-analysis (I^2^ = 92.57%, *p* < 0.001; Figure 2). Meta-analysis found significant linear relationships between feed intake and NPR (*P* = 0.016; R^2^ = 4%) and processing methods (*P* = 0.002; R^2^ = 14%) as described in Table 3. There was no significant linear relationship between feed intake and broiler strain (*P* = 0.091; R^2^ = 0%) and cassava form (*P* = 0.067; R^2^ = 5%) in response to cassava intervention. There is minimal evidence of publication bias as displayed in our funnel graphs (Supplementary Figure S1).

**Table 2:** Subanalysis comparing the effects of cassava on growth traits in broiler chickens.

**Covariates**	**Feed intake**			**FCR**			**ADG**		
	**SMD**	**95% CI**	***p*–Value**	**SMD**	**95% CI**	***p*–Value**	**SMD**	**95% CI**	***p*–Value**
Cassava form									
CRM	−0.33	−0.70, 0.04	0.082	0.98	0.44, 1.52	< 0.001	−1.97	−2.47, −1.47	< 0.001
CRM + ES	−1.29	−2.49, −0.09	0.034	0.47	−3.69, 2.74	0.773	−3.11	−4.81, −1.42	< 0.001
CPM	−0.24	−0.54, 0.61	0.118	−0.32	−1.39, 0.76	0.561	−0.83	−2.29, 0.63	0.266
CPM + CLM	0.09	−0.07, 0.25	0.271	1.77	1.12, 2.42	< 0.001	−1.59	−2.17, −1.01	< 0.001
WFCPM	0.62	0.47, 0.77	< 0.001	−0.26	−0.65, 0.13	0.191	0.66	0.37, 0.95	< 0.001
SSFCRM	0.18	−0.11, 0.47	0.216	0.54	0.05, 1.03	0.032	−0.33	−0.60, −0.06	0.017
CRSM	−0.42	−0.68, −0.16	0.001	−0.02	−0.54, 0.50	0.994	−0.38	−1.08, 0.31	0.282
CRWM	0.15	−0.42, 0.72	0.616	1.39	−0.08, 2.85	0.063	−1.51	−3.58, 0.56	0.152
WFCRM	0.55	0.32, 0.77	< 0.001	3.57	2.90, 4.25	< 0.001	−2.83	−3.45, −2.22	< 0.001
CPM + ES	−0.20	−0.50, 0.09	0.181	2.51	0.25, 4.76	0.029	−2.67	−5.08, −0.26	0.030
SSFCPM	0.52	−0.78, 1.82	0.433	1.21	0.56, 1.86	< 0.001	−1.21	−1.68, −0.75	< 0.001
Broiler strains									
Cobb	−0.17	−0.68, 0.35	0.527	0.74	0.12, 1.40	0.019	−1.45	−2.21, −0.69	< 0.001
Ross	0.31	−0.31, 0.92	0.331	1.57	0.88, 2.26	< 0.001	−1.07	−1.39, −0.74	< 0.001
Anak	−0.04	−0.29, 0.20	0.721	2.04	1.28, 2.79	< 0.001	−2.28	−3.00, −1.57	< 0.001
Arbor Acres	−0.59	−1.08, −0.09	0.021	0.98	−0.08, 2.05	0.071	−1.50	−2.77, −0.23	0.020
Marshal	−0.03	−0.24, 0.18	0.781	0.17	−0.91, 1.25	0.754	−2.48	−3.34, −1.61	< 0.001
Inclusion (%)									
4–10	0.33	0.02, 0.64	0.040	0.56	−0.43, 1.46	0.270	−0.45	−1.59, 0.68	0.345
11–20	0.06	−0.10, 0.22	0.396	0.80	0.38, 1.23	< 0.001	−0.77	−1.18, −0.36	< 0.001
21–30	−0.07	−0.35, 0.21	0.617	1.14	0.37, 1.91	0.004	−1.48	−2.34, −0.62	< 0.001
31–40	−0.09	−0.68, 0.50	0.822	0.93	−0.06, 1.92	0.066	−2.07	−2.71, −1.43	< 0.001
41–50	−0.30	−1.06, 0.47	0.445	2.21	0.60, 3.82	0.007	−3.59	−4.79, −2.39	< 0.001
51–60	−0.52	−1.62, 0.57	0.349	1.88	1.05, 2.71	< 0.001	−3.28	−4.26, −2.29	< 0.001
NPR									
< 10	−0.16	−0.45, 0.13	0.274	0.20	−0.45, 0.85	0.553	−2.04	−2.74,−1.34	< 0.001
10	0.10	−0.18, 0.37	0.493	1.65	1.28, 2.03	< 0.001	−1.62	−1.96, −1.29	< 0.001
>10	−0.61	−1.07, −0.15	0.010	1.02	0.03, 2.01	0.043	−1.58	−2.78, −0.38	0.010
Processing methods									
Drying	−0.24	−0.45, −0.03	0.024	0.81	0.40, 1.22	< 0.001	−1.60	−2.01, −1.18	< 0.001
Drying + ES	−0.68	−1.35, −0.02	0.043	1.22	−0.51, 2.95	0.168	−2.84	−4.27, −1.42	< 0.001
WF + Drying	0.59	0.47, 0.71	< 0.001	2.65	1.72, 3.58	< 0.001	−2.00	−2.91, −1.10	< 0.001
SSF + Drying	0.38	−0.41, 1.18	0.345	0.95	0.48, 1.41	< 0.001	−0.87	−1.26, −0.48	< 0.001

SMD, standardized mean difference; CI, confidence interval; CRM, cassava root meal; ES, enzyme supplementation; CPM, cassava peel meal; CLM, cassava leaf meal; WFCPM, wet fermented cassava peel meal; SSFCRM, solid state fermented cassava root meal; CRSM, cassava root sievate meal; CRWM, cassava root waste meal; WFCRM, wet fermented cassava root meal; SSFCPM, solid state fermented cassava peel meal.

**Table 3:** Relationships between growth traits and covariates in response to cassava intervention.

**Outcomes**	**Moderators**	**Model estimates**		
		**Q_M_**	***p*-value**	**R^2^ (%)**
Feed intake	Cassava form	18.10	0.053	5
	Inclusion level	7.75	0.459	0
	Broiler strains	8.03	0.091	0
	NPR	8.26	0.016	4
	Processing methods	14.60	0.002	14
FCR	Cassava form	29.60	0.001	20
	Inclusion level	4.45	0.487	0
	Broiler strains	7.85	0.097	4
	NPR	8.21	0.017	8
	Processing methods	8.89	0.031	6
ADG	Cassava form	17.40	0.067	8
	Inclusion level	29.4	1.69e-05	24
	Broiler strains	6.44	0.168	2
	NPR	0.84	0.657	0
	Processing methods	3.00	0.395	0

ADG, average daily gain; FCR, feed conversion ratio; NPR, number of broiler per replicate; Q_M_, test of moderators; R^2^, amount of heterogeneity accounted for by the moderators; p, probability.

### Feed conversion ratio

**Figure 3:**
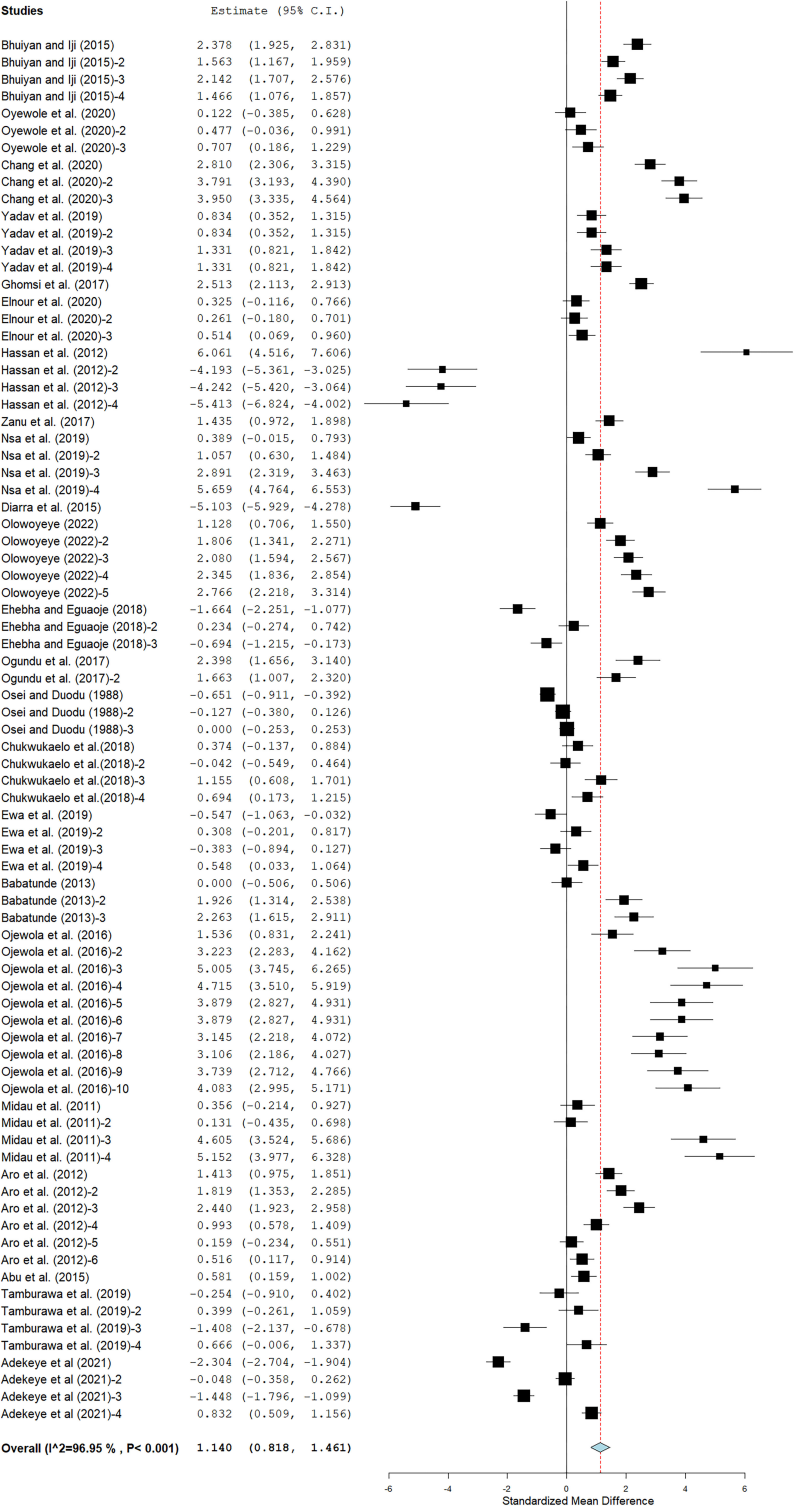
MEB of the impact of cassava on FCR of broiler chickens. The thick vertical line is the line of no effect. Plots to the left of the solid vertical line show a decrease in FCR, whereas plots to the right show an increase in FCR.

Cassava intervention had a large positive effect on FCR (SMD = 1.14; 95% CI 0.82, 1.46; Figure 3). Table 2 indicates that CRM, a blend of CLM and CPM, cassava root waste meal (CRWM), WFCPM, CPM + ES, and solid-state fermented CPM (SSFCPM) had large positive effects on FCR. Broiler strain had a small to a large positive effect on FCR. However, the inclusion level had a moderate to a large positive effect on FCR. The magnitude of effect size was highest in studies that included more than 10 broilers in each replicate, but lowest in studies that used 10 broilers. Results show that processing methods had a large impact on FCR. There is a presence of significant heterogeneity (I^2^ = 96.95%, *p* < 0.05) among studies that evaluated the benefits of cassava form on FCR in broiler chickens (Figure 3). There are significant linear relationships between FCR and cassava form (*P* = 0.001; R^2^ = 20%), NPR (*P* = 0.017; R^2^ = 8%), and processing methods (*P* = 0.031; R^2^ = 6%) in response to cassava intervention (Table 3). There was no significant linear relationship between chicken strain and FCR (*P* = 0.097; R^2^ = 0%) in response to cassava treatment interaction. There is small evidence of publication bias as the funnel plots were nearly symmetrical (Supplementary Figure S2). The Rosenberg Nfs for the database is 18,759, which is more than 45 times above the threshold of 415 (5 × n = 81 + 10) needed to consider the effect size robust.

### Average daily gain

**Figure 4:**
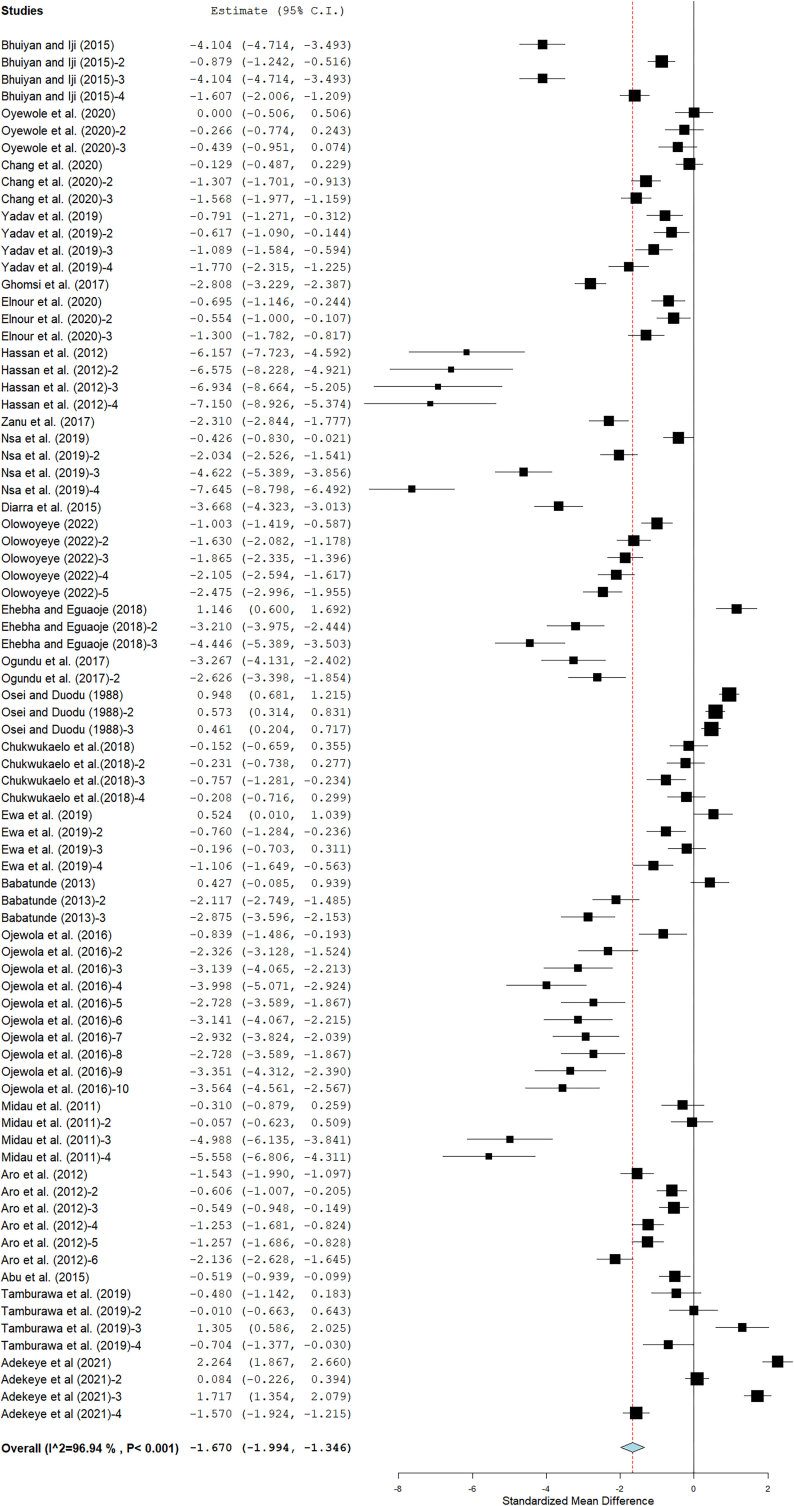
MEB of the effect of cassava in broiler chicken diets on ADG. The thick vertical line is called the line of no effect. Plots to the left of the line of no effect show a reduction in ADG, whereas plots to the right of the line of no effect suggest an increase in ADG.

The impact of cassava on the ADG of broiler chickens was large and negative (SMD = −1.67; 95% CI −1.99, −1.35; Figure 4). Results of the subgroup analysis of the impacts of studied covariates on ADG are presented in Table 2. Mean effect sizes for CRM, CRM + ES, CPM, CPM + CLM, CRWM, WFCRM, CRM + ES, and SSFCPM were −1.97, −3.11, −0.83, −1.59, −1.51, −2.83, −2.67, and −1.21, indicating that aspects of cassava form had large and negative impacts on ADG. Interestingly, the mean effect size for WFCPM was 0.66, with 95% CI 0.37–0.95, implying that WFCPM had a moderate and positive effect on ADG in broiler chickens. The mean effect sizes for broiler strains and inclusion levels were large and negative. NPR had a large and negative impact on the ADG of broiler chickens. Results suggested that mean effect estimations were −1.60, −2.84, −2.00, and −1.26 for sun-drying, sun-drying + ES, wet fermentation + sun-drying, and solid-state fermentation + sun-drying, respectively, implying that processing methods had large and negative effects on ADG. Figure 4 shows evidence of large significant heterogeneity among studies that assessed ADG. Meta-regression found low effect for inclusion level as a covariate (*P* < 0.001; R^2^ = 24%) for ADG in broiler chickens (Table 3). Funnel plots obtained in this study were near asymmetrical (Supplementary Figure S3). However, the Rosenberg Nfs for the database is 18,172, which is 43 times greater than the value of 415 (5 × n = 81 + 10) needed to alter the significant effect of cassava on ADG in broilers.

## Discussion

To the best of our knowledge, this is the first meta-analysis to assess the effects of diets with and without cassava on the performance characteristics of broiler chickens. Cassava is rich in energy and fiber but deficient in methionine and lysine (4, 7). It is widely cultivated in most tropical regions of the world. Our pooled results showed that cassava had a large reduction effect on the growth performance traits of broiler chickens. The observed negative performance characteristics in broiler chickens fed cassava in this study could be attributed to the inhibitory effect of cyanide on the cytochrome oxidase, an enzyme that plays an essential role in the mitochondrial aerobic energy metabolism of the respiratory chain (6, 7). The results of this study are inconsistent with the findings of Aladi et al. (5) who discovered improved growth performance traits in broiler chickens fed varying levels of fermented cassava. This disparity may be due to the reflex of partial replacement of maize with cassava. Our results suggested minimal evidence of publication bias. However, this is not an issue as a relatively large number of unpublished studies would be required to change the statistically significant effects of cassava intervention (26).

## Explanatory moderator variables

### Cassava form

This study found that cassava form was a limiting factor and accounted for 20% of the between-study variance in FCR. The amount of heterogeneity not explained by the mixed-effects model could be linked to variables such as the age of the chicken, amount of cyanide present in cassava, variety of cassava used, sex of chicken, and diet composition that were not tested in this study due to insufficient data. High dietary fiber limits the transit time of feed in the digestive tracts of chickens (29). The moderately lower feed intake in the subgroup fed CRSM when compared to controls implies that the low ability of broiler chickens to digest the high fiber present in CRSM diets causes a gut-fill sensation. In contrast, the moderately increased feed intake in the group offered WFCPM and WFCRM when compared to controls indicates the enhanced ability of wet fermentation to reduce cyanide levels in CPM and CRM. Two possible mechanisms contributing to the increased feed intake in broiler chickens fed WFCPM and WFCRM are the introduction of microbial linamarase to the cassava and cell-wall-degrading enzymes that permit contact between the compartmentally separated linamarin and endogenous limamarase. The inclusion of sun-dried CRM in broiler chicken diets in this meta-analysis resulted in poor FCR but improved enzyme supplementation. Similar studies have been reported by Saleh et al. (30) and Acamovic (31) who found reduced FCR in broiler chickens fed enzyme-biodegraded CRM. Our results show that birds fed WFCPM diets had higher ADG than controls, suggesting that WFCPM-based diets support muscle tissue accretion. In addition, the moderately higher ADG of broiler chickens fed WFCPM when compared to those offered SSFCPM is perhaps attributed to the better capacity of wet fermentation to detoxify cyanogenic glucosides in CPM than solid-state fermentation.

### Broiler chicken strains and number per replication

Our results suggest that cassava form had large and negative effects on ADG in all the broiler strains analyzed in the current meta-analysis. Results found no significant linear relationships between broiler strain and response variables. However, there was a small effect of NPR as a covariate for feed intake and FCR, and no more than 12% of the sources of heterogeneity were explained by NPR. The subgroup analysis revealed that broiler chickens from studies that included < 10 birds in each replicate had similar feed intake and FCR with controls, but these results did not translate to improved ADG. No similar studies in broiler chickens were found in the literature to compare with our results. As a result, there is a need for research in this direction.

### Inclusion level

Inclusion level is a significant predictor of ADG. Broilers fed low levels of cassava form (4–10%) had comparable FCR and ADG values with controls, suggesting that the inclusion of low levels of cassava in the chicken feed had beneficial effects on the growth variables of broiler chickens. Birds have a genetically defined requirement for nutrients (2). The observed significantly higher feed intake in broiler chickens fed low inclusion levels of cassava when compared to controls may be an attempt by the birds to consume more feed in order to meet their requirements for the limiting nutrients in cassava diets (7). The results of this study also showed reduced growth traits in broiler chickens fed higher inclusion levels of cassava. Our results also suggest that dilution of chicken feed with material low in protein and high in fiber is most likely to result in suboptimal performance. The fact that broiler chickens fed low inclusion levels of cassava compared favorably with the controls implies that this may be the level that supports optimal growth characteristics.

### Processing methods

The use of cassava in animal feed is limited by its low protein level, unbalanced amino acid profile, dusty nature of the dry meals, and the presence of anti-nutritional factors (6). However, these constraints can be moderately remedied *via* adequate processing methods. Our results showed a small effect for processing methods as a covariate and explained approximately 20% of the intervention effect. This confirmed the previous reports that adequate processing methods can improve the utilization of cassava in broiler chicken diets (6, 32). Our results suggested that a blend of wet fermentation and sun-drying methods were effective in increasing the feed value of cassava. This might be attributed to the action of microbial enzymes in enabling linamarin to have contact with its hydrolytic enzyme (linamarase), resulting in hydrolysis and subsequent removal of the breakdown products (33). The significantly reduced feed intake of broiler chickens fed sundried cassava indicates the low ability to dry as a processing method to enhance the feeding quality of cassava in broiler chickens.

## Limitations and strengths of the meta-analysis

This meta-analysis was limited to research on broiler chickens alone and may not apply to other poultry species. Differences in age, strain, and sex of broiler chickens may pose a limitation to this data synthesis. Despite these limitations, the strength of this study includes the use of systematic and explicit methods to identify, select, critically appraise, and analyse published trials on the effect of the replacement of maize with cassava on the growth performance of broiler chickens. This study also showed the potential for cassava to replace maize in broiler chicken diets. This research also sets the steps for standardized experimental designs on the use of cassava to replace maize in broiler chicken diets in the future.

## Conclusion and future research direction

Results showed that cassava root and cassava peel meals were the most cassava form used in the broiler chicken industry. The results of this meta-analysis demonstrated that the addition of cassava types to chicken diets did not improve the growth characteristics of broiler chickens. In contrast, broilers fed wet fermented cassava peel meal had improved growth performance traits, which could be attributed to the potential of microbial enzymes implicated in wet fermentation to enhance the nutritional value of cassava by increasing their crude protein content and decreasing their crude fiber and cyanide contents. The future research trends are to determine the optimum replacement level of maize for cassava in broiler chickens. The meta-analysis showed that studied covariates explained most of the sources of heterogeneity. Few studies evaluated the effect of strains on performance traits of broiler-fed cassava diets; hence future research efforts should be directed in this area.

## Data availability statement

The original contributions presented in the study are included in the article/Supplementary material, further inquiries can be directed to the corresponding author/s.

## Author contributions

IO: conceptualization, data analysis, and visualization. IO and CM: writing—manuscript preparation, review, and editing. Both authors have read and agreed to the published version of the manuscript.

## Conflict of interest

The authors declare that the research was conducted in the absence of any commercial or financial relationships that could be construed as a potential conflict of interest.

## Publisher's note

All claims expressed in this article are solely those of the authors and do not necessarily represent those of their affiliated organizations, or those of the publisher, the editors and the reviewers. Any product that may be evaluated in this article, or claim that may be made by its manufacturer, is not guaranteed or endorsed by the publisher.
