# Supplementary material for: Meta-analysis of substitution value of maize with cassava (Manihot esculenta Cratnz) on growth performance of broiler chickens
Source: Front Vet Sci. 2022 Nov 14;9:997128. doi: 10.3389/fvets.2022.997128 (PMC9701717; doi:10.3389/fvets.2022.997128)
Supplement: Supplementary file 2 [file Table_2.docx]

**Supplementary Table S2** Characteristics of studies included in the meta-analysis

| **Authors** | **Country** | **Continent** | **RD (day)** | **Covariates** | | | | | **Response variables** |
| --- | --- | --- | --- | --- | --- | --- | --- | --- | --- |
|  |  |  |  | **Cassava products** | **Inclusion (%)** | **Strain** | **Processing methods** | **NPR** |  |
| Osei and Duodu (1988) | Ghana | Africa | 1-42 | WFCPM | 0 - 15 | Cobb | WF + Drying | 20 | FI, ADG, FCR |
| Midau et al. (2011) | Nigeria | Africa | 1-56 | CPM + ES | 0 - 48 | Anak | Drying + ES | 8 | FI, ADG, FCR |
| Aro et al. (2012) | Nigeria | Africa | 1-56 | SSFCPM | 0 - 60 | Ross | SSF + Drying | 10 | FI, ADG, FCR |
| Hassan et al.(2012) | Nigeria | Africa | 1-56 | CRM | 0 - 57.85 | Marshal | Drying | 6 | FI, ADG, FCR |
| Babatunde (2013) | Fiji | Oceania | 1-46 | CRWM | 0 - 30 | Cobb | Drying | 10 | FI, ADG, FCR |
| Abu et al. (2015) | Nigeria | Africa | 1-49 | CPM + CLM | 0 - 20 | Arbor acres | Drying | 15 | FI, ADG, FCR |
| Diarra et al.(2015) | Samoa | Oceania | 1-42 | CRM + ES | 0 - 34.03 | Cobb | Drying + ES | 4 | FI, ADG, FCR |
| Bhuiyan and Iji (2015) | Australia | Oceania | 1-21 | CRM + ES | 0 - 51 | Cobb | Drying + ES | 10 | FI, ADG, FCR |
| Ojewola et al. (2016) | Nigeria | Africa | 1-56 | WFCRM | 0 - 58.6 | Anak | WF + Drying | 10 | FI, ADG, FCR |
| Ghomsi et al.(2017) | Cameroon | Africa | 1-46 | CPM | 0 - 4 | Arbor acres | Drying | 12 | FI, ADG, FCR |
| Ogundu et al.(2017) | Nigeria | Africa | 1-56 | CRM | 0 - 39.93 | Anak | Drying | 8 | FI, ADG, FCR |
| Zanu et al. (2017) | Ghana | Africa | 1-42 | CRM | 0 - 58 | Cobb | Drying | 15 | FI, ADG, FCR |
| Chukwukaelo et al.(2018) | Nigeria | Africa | 1-56 | SSFCRM | 0 - 31 | - | SSF + Drying | 10 | FI, ADG, FCR |
| Ehebha and Eguaoje (2018) | Nigeria | Africa | 1-56 | CPM | 0 - 30 | Anak | Drying | 10 | FI, ADG, FCR |
| Ewa et al. (2019) | Nigeria | Africa | 1-49 | CRSM | 0 - 20 | Anak | Drying | 10 | FI, ADG, FCR |
| Tamburawa et al. (2019) | Nigeria | Africa | 1-56 | CRM | 0 - 36.05 | Marshal | Drying | 6 | FI, ADG, FCR |
| Yadav et al.(2019) | USA | NA | 1-42 | CRM | 0 - 50 | Cobb | Drying | 6 | FI, ADG, FCR |
| Nsa et al. (2019) | Nigeria | Africa | 1-42 | CRM | 0 - 60 | Amos | Drying | 16 | FI, ADG, FCR |
| Oyewole et al.(2020) | Nigeria | Africa | 1-56 | CRM | 0 - 31.52 | - | Drying | - | FI, ADG, FCR |
| Chang et al.(2020) | Australia | Oceania | 1-35 | CRM + ES | 0 - 44.57 | Ross | Drying + ES | 10 | FI, ADG, FCR |
| Elnour et al.(2020) | Sudan | Africa | 1-35 | CRM | 0 - 40 | Ross | Drying | 10 | FI, ADG, FCR |
| Olowoyeye (2022) | Nigeria | Africa | 1-42 | CPM + CLM | 0 - 29.12 | Marshal | Drying | 10 | FI, ADG, FCR |
| Adekeye et al. (2021) | Nigeria | Africa | 1-42 | CPM | 0 - 30 | Arbor acres | Drying | 16 | FI, ADG, FCR |

*NA* North America*, RD* rearing durations*, CRM* cassava root meal, *ES* enzyme supplementation, *CPM* cassava peel meal, *CLM* cassava leaf meal, *WFCPM* wet fermented cassava peel meal, *WFCRM* wet fermented cassava root meal, *SSFCRM* solid state fermented cassava root meal, *SSFCPM* solid state fermented cassava peel meal, *CRSM* cassava root sievate meal, *CRWM* cassava root waste meal, *NPR* number per replicate, *FI* feed intake, *ADG* average daily gain, *FCR* feed conversion ratio,

**Bibliography of included studies for the meta-analysis**

Abu OA, Olaleru IF, Oke TD, Adepegba VA, Usman B. Performance of broiler chicken fed diets containing cassava peel and leaf meals as replacements for maize and soya bean meal. Int J Sci and Tech. (2015) 4: 169-173.

Adekeye AB, Amole TA, Oladimeji SO, Raji AA, Odekunle TE, Olasusi O, et al. Growth performance, carcass characteristics and cost benefit of feeding broilers with diets containing high quality cassava peel (HQCP). Afr J Agric Res. (2021) 17: 448 - 455. doi: 10.5897/AJAR2020.15237

Aro SO, Agbede JO, Dairo OO, Ogunsote E, Aletor VA. Evaluation of fermented cassava tuber wastes in broiler chickens feeding. Archiva Zootechnica (2012)15: 49-60.

Babatunde,BB. Effect of feeding cassava wastes on the performance and meat quality of broiler chickens. Mal J Anim Sci. (2013) 16: 63-73.

Bhuiyan MM, Iji PA. Energy value of cassava products in broiler chicken diets with or without enzyme supplementation. Asian-Austral J Anim Sci. (2015) 28: 1317-1326. doi:10.5713/ajas.14.0915

Chang EP, Abdallh ME, Ahiwe EU, Mbaga S, Zhu ZY, Fru-Nji F, Iji PA. Replacement value of cassava for maize in broiler chicken diets supplemented with enzymes. Asian-Australas J Anim Sci. (2020) 33: 1126-1137. doi:10.5713/ajas.19.0263

Chukwukaelo AK, Aladi NO, Okeudo NJ, Obikaonu HO, Ogbuewu IP, Okoli IC. Performance and meat quality characteristics of broilers fed fermented mixtures of grated cassava roots and palm kernel cake as replacement for maize. Trop Anim Hlth and Prod. (2018) 50: 485-493. [doi](https://doi): 10.1007/s11250-017-1457-7

Diarra SS, Sandakabatu D, Perera D, Tabuaciri P, Mohammed U. Growth performance and carcass yield of broiler chickens fed commercial finisher and cassava copra meal-based diets. J Appld Anim Res. (2015) 43: 352-356. doi: 10.1080/09712119.2014.978774

Ehebha ETE, Eguaoje AS. Growth performance characteristics of broiler chickens fed graded levels of sundried cassava (Manihot esculenta) peel meal based diet. Asian J Adv Agric Res. (2018) 6:1-7. doi: 10.9734/AJAAR/2018/41079

Elnour Z, Babiker M, Habib A. Effect of different levels of cassava roots on growth performance, carcass traits and meat quality of broiler chickens. Int J Livestock Res. (2020) 10: 20-28. doi: doi: 10.5455/ijlr.20200627105321

Ewa UE, Kalu NC, Adedokun OO, Oka U, Onabanjo RS, Ezike JC. Replacement of maize with graded dry cassava sievate in broiler chicken ration. Nig J Anim Sci. (2019) 21: 291-300.

Ghomsi MO, Doube TB, Etchu KA, Fotso JM, Tchakounte J, Mongo GB, et al. Performance of broiler chickens fed on cassava peels and rice bran as energy substitute to maize. SOJ Vet Sci. (2017) 3: 1-5. doi: 10.15226/2381-2907/3/5/00145

Hassan AM, Tamburawa MS, Alponsus C, Yusuf JH. Studies on growth, organs weight and haematological parameters of broiler chicken fed graded level of sun dried cassava root meal. Bayero J Pure and Appld Sci. (2012) 5: 98 – 102. doi:10.4314/bajopas.v5i1.18

Midau A, Augustine C, Yakubu B, Yahaya SM, Kibon A, Udoyong AO Performance of broiler chicken fed enzyme supplemented cassava peel meal-based diets. Int J Agr Sustain. (2011) 3:1–4

Nsa EE, Ukoha OA, Agida CA. Bio-economics of feeding cassava root meal based diets to broiler finisher chickens. Nig J Anim Prod. (2019) 46: 110 - 116. doi: 10.51791/njap.v46i4.297

Ogundu EC, Ekpo SJ, Ukpanah AU, Essien AC, Iboro OF. Performance and carcass quality of broiler placed on provitamin a cassava and sweet cassava-based diets. Asian J. Agric. and Food Sci. (2017) 5: 169-175.

Ojewola GS, Ebele EA, Olojede AO. Biologic and economic performance of broiler chickens fed cassava root meal “garri” as a substitute for maize. IOSR J Agric and Vet Sci (IOSR-JAVS) (2016) 9: 10-15. doi: 10.9790/2380-0910011015

Olowoyeye JC. Economics of production of broiler chickens fed cassava peel leaf meal (ratio 9:1) mix as replacement for maize. Int J Innovat Sci and Res Tech. (2022) 7: 377-381. doi:10.5281/zenodo.6392248

Osei SA, Duodu S. Effect of fermented cassava peel meal on the performance of broilers. Brit. Poult. Sci. (1988) 29: 671-675. doi: 10.1080/00071668808417093

Oyewole NO, Akinyele SA, Ogunsipe MH. Effect of cassava pulp substituting maize on the growth performance and haemato-biochemical attributes of broiler chickens. Livestock Res. for Rural Dev. (2020) 32:1-6. Retrieved from http://www.lrrd.org/lrrd32/11/moogu32174.html

Tamburawa MS, Abubakar Z, Salisu N, Wudil AA, Hassan AM, Ibrahim U, et al. Effect of dietary utilization of cassava root (Manihot esculenta) meal on growth performance and carcass characteristics of broiler chickens at finisher phase. Nig J Anim Sci and Tech. (2019) 2: 63 – 71.

Zanu HK, Azameti MK, Asare D. Effects of dietary inclusion of cassava root flour in broiler diets on growth performance, carcass characteristic and haematological parameters. Int J Livestock Prod. (2017) 8: 28-32. doi: 10.5897/IJLP2015.0222

Yadav S, Mishra B, Jha R. Cassava (Manihot esculenta) root chips inclusion in the diets of broiler chickens: effects on growth performance, ileal histomorphology, and cecal volatile fatty acid production. Poult Sci. (2019) 98: 4008–4015. doi: 10.3382/ps/pez143
