# Supplementary figures and images for: Meta-analysis of substitution value of maize with cassava (Manihot esculenta Cratnz) on growth performance of broiler chickens
Source: Front Vet Sci. 2022 Nov 14;9:997128. doi: 10.3389/fvets.2022.997128 (PMC9701717; doi:10.3389/fvets.2022.997128)

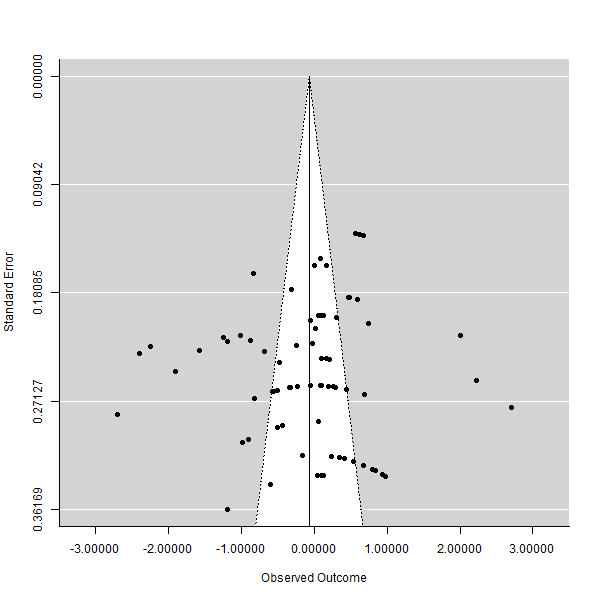


**Supplementary Figure 1** Funnel graph of the impact of cassava on feed intake in broilers.

Supplement: Supplementary file 4 [file Data_Sheet_1.doc]

**
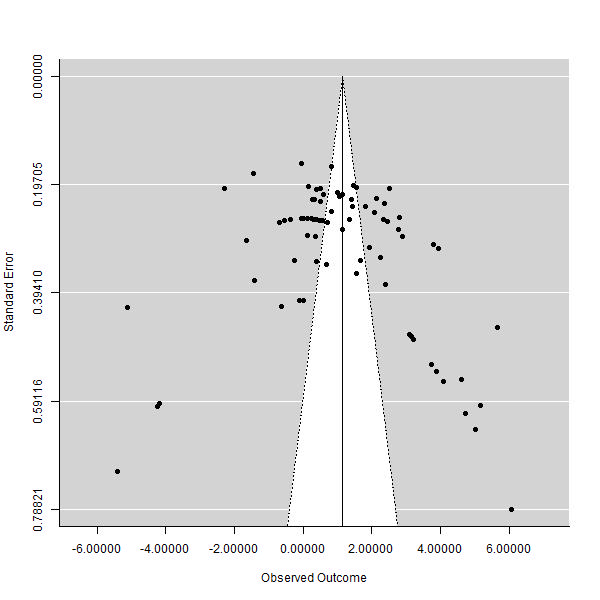
**

**Supplementary Figure 2** Funnel plots of the influence of cassava on FCR in broiler chickens.

Supplement: Supplementary file 5 [file Data_Sheet_2.doc]
