# Supplementary material for: Meta-analysis of substitution value of maize with cassava (Manihot esculenta Cratnz) on growth performance of broiler chickens
Source: Front Vet Sci. 2022 Nov 14;9:997128. doi: 10.3389/fvets.2022.997128 (PMC9701717; doi:10.3389/fvets.2022.997128)
Supplement: Supplementary file 6 [file Data_Sheet_3.doc]

**
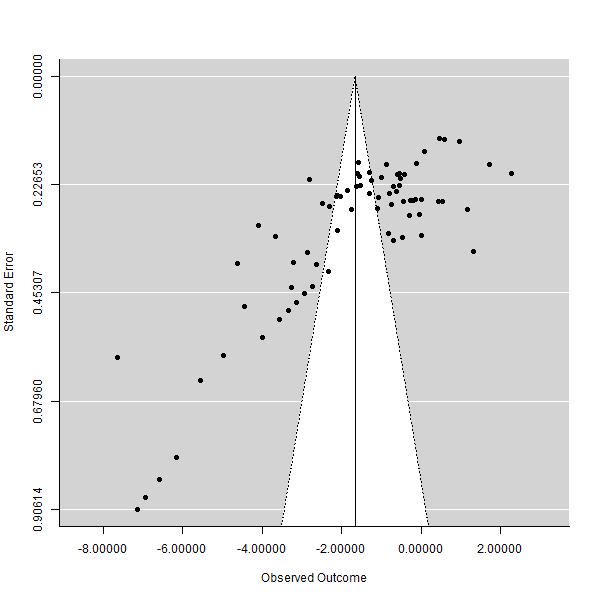
**

**Supplementary Figure 3** Funnel plots of the impact of cassava intervention on ADG in broiler chickens.
